# Performance of Computed Tomography of the Kidneys, Ureter and Bladder in Non-Calculus Diagnoses: A Comparative Review of Non-Enhanced with Intravenous Contrast-Enhanced Imaging

**DOI:** 10.3390/diagnostics15141731

**Published:** 2025-07-08

**Authors:** Alexander T. O’Mahony, Michael G. Waldron, David J. Ryan, Brian Carey, Sahil Shet, Eid Kakish, Patrick O’Regan, David Glynn, Josephine Barry, Owen J. O’Connor, Michael M. Maher

**Affiliations:** 1 Department of Radiology, Cork University Hospital, T12DC4A Cork, Ireland; alexlocum@gmail.com (A.T.O.); david.ryan109@ucc.ie (D.J.R.); bcarey@umail.ucc.ie (B.C.); david.glynn@umail.ucc.ie (D.G.); josephine.barry@hse.ie (J.B.); oj.oconnor@ucc.ie (O.J.O.); m.maher@ucc.ie (M.M.M.); 2 Department of Radiology, School of Medicine, Brookfield Health Complex, University College Cork, T12Ak54 Cork, Ireland; ekakish@ucc.ie

**Keywords:** urinary calculi, non-contrast enhanced CT KUB, contrast-enhanced CT KUB, diagnostic yield, effective radiation dose

## Abstract

**Background/Objectives**: Non-enhanced computed tomography of the kidneys, ureters and bladder (NECT KUB) is the initial imaging modality for suspected nephroureterolithiasis. However, for alternative diagnoses, NECT may not be the ideal technique. Our institution changed the protocol for this cohort from NECT to intravenous contrast-enhanced CT (CECT) KUB. We aimed to retrospectively compare the rate of alternative diagnosis seen and the rates of calculus detection in CECT versus NECT KUB as a means of assessing performance. Our secondary aim was to compare the radiation dose between CECT and NECT KUB. **Methods**: Patients referred from the emergency department with suspected nephroureterolithiasis who underwent NECT and CECT KUB over two years were included. Key performance metrics included calculus detection rate, alternative findings, and negative studies. The metrics were compared between genders and age groups. Categorical variables were analysed using Chi-squared or Fisher’s Exact Test and continuous with T-testing. **Results**: A total of 423 patients had CT KUB imaging (209 NECT, 214 CECT). The incidence of alternative findings in the NECT group was 23% and 40% in CECT (*p* < 0.001). There were 48 findings (13 major, 11 moderate and 24 minor) in NECT studies and 85 findings (23 major, 43 moderate and 19 minor) in CECT (*p* < 0.001). Major diagnoses ranged from acute emergencies to more indolent findings, including suspicious nodules/masses. The calculus detection rate (NECT 56%, CECT 54%, *p* = 0.643) and negative studies (NECT 28%, CECT 22%, *p* = 0.168) did not significantly differ between protocols. CECT had a mean effective dose of 8.71 ± 2.58 mSv representing 2.4 times the exposure of NECT (*p* < 0.001). **Conclusions**: CECT is associated with a greater alternative diagnosis rate with similar calculus detection rates compared to NECT KUB, suggesting superior performance. However, CECT exposes patients to significantly greater levels of ionizing radiation.

## 1. Introduction

The symptoms of nephroureterolithiasis (NU) are a common aetiology of emergency department (ED) presentations. Historically, the plain radiograph and intravenous pyelogram (IVP) were first-line investigations in suspected stone disease. In 1995, a paradigm shift occurred with the introduction of non-enhanced computed tomography of the kidneys, ureters and bladder (NECT KUB) [1]. NECT KUB had a higher sensitivity and specificity for stone detection than IVP whilst not requiring the administration of iodinated contrast and, importantly, could also detect alternative diagnoses to account for the patient’s presentation [1,2]. Not surprisingly, NECT KUB was embraced by most professional societies and is now the international standard of care where suspicion of symptomatic nephroureteric calculi exists [3,4].

As NECT KUB is a highly specialised examination tailored to detect calculi, the acquisition parameters and absence of both oral and IV (intravenous) contrast media do not permit the confident exclusion of pathologies in the vasculature, hollow and solid viscera. Potentially devastating diagnoses can be omitted in non-enhanced studies. An estimated 1/3rd of renal cell cancers (RCC) may be missed when CT imaging is unenhanced, and all early RCC diagnoses are highly challenging [5,6]. The detection of alternative hyper-vascular abdominal malignancies (e.g., pancreatic cancer and hepatocellular carcinoma) has a reported sub-standard sensitivity and specificity without contrast enhancement [7,8]. Furthermore, pathologies, including renal infarct, pyelonephritis, acute cholecystitis and early acute appendicitis, have been reported as undetectable prior to IV contrast administration [9,10,11]. While these clinically relevant alternative findings are proportionally low, their significance is high and may frequently be underdiagnosed in acute settings, resulting in delayed and more complicated presentations with potential medico-legal implications.

The Royal College of Radiology (RCR) has set standards to aid with institutional, regional and national audits for NECT KUB. A calculus detection rate (CDR) of 44–64% and alternative diagnoses of 6–18% are the key performance metrics indicating the appropriateness of this investigative technique [1,3,4,12]. Numerous studies and regional audits have reported a persistently low CDR within specific groups. Females have shown CDR’s as low as 26% with no substantial difference in their alternative findings when studies are non-enhanced. Furthermore, this has been reported in geriatric males with no previous history of NU [12,13,14]. The poor performance, along with anecdotal evidence in a multitude of radiological departments over the limitations of CT KUB and the potential lack of awareness of these limitations by referring clinicians, is concerning.

All CT imaging carries the risks associated with ionising radiation exposure, and the exponential rise in its use on a global level is a serious issue. Contrast-enhanced computed tomography (CECT) is associated with higher effective doses (EffD) of ionising radiation than NECT, a fact that should not be neglected [15]. However, it is essential to understand the limitations of specific imaging techniques and make decisions that balance the risks and benefits of the technique in question. The hypothesis of this study was that CECT, despite its higher EffD, would allow for the detection of a greater number of alternative diagnoses in comparison to NECT.

The primary aim of this study was to retrospectively compare three key performance metrics: CDR, alternative radiological findings, and negative studies between NECT and CECT KUB imaging after a change in institutional radiological protocol whereby first-line investigation for suspicion of NU moved to contrast-enhanced CT imaging from ED referrals.

## 2. Materials and Methods

### 2.1. Study Design

Institutional review board approval was obtained for this single-centre retrospective study, which took place in a tertiary care centre. Patients who attended the emergency department (ED) with suspected nephrolithiasis and where subsequent CT KUB imaging was requested by the ED physician were included.

In 2019 the institutional protocol of CT KUB requests by ED physicians for suspected nephrolithiasis changed from NECT KUB to CECT KUB. Two full calendar years were chosen to compare imaging techniques. No changes were made to the patient selection criteria following the change in protocol. Patients with suspected nephrolithiasis simply underwent CECT from 2019 onwards, whereas NECT was utilised prior to this. Therefore, those in 2018 who presented to the ED with suspected calculi underwent NECT KUB, while in 2020 patients received CECT KUB.

### 2.2. Population Sample

**Figure 1:**
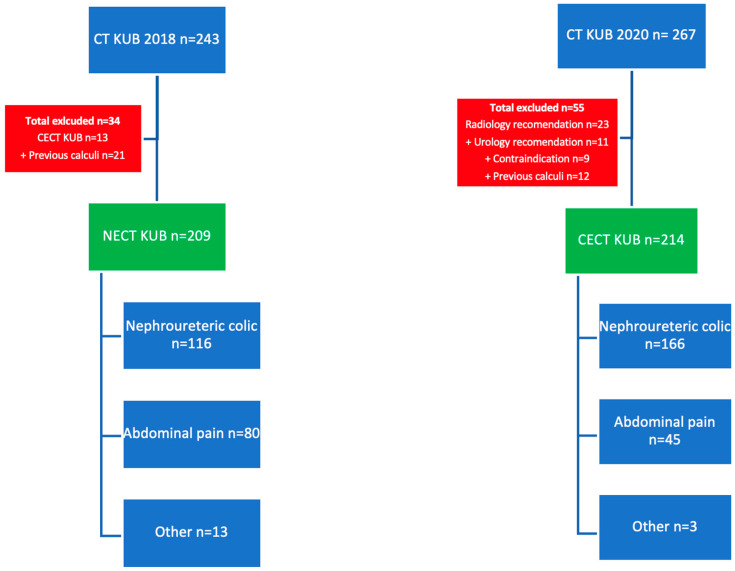
Flowchart demonstrating the selection of patients from the non-enhanced (2018 cohort) and contrast enhanced (2020 cohort) CT KUB groups.

In total, 243 patients presenting with suspected NU underwent NECT KUB during 2018. In 13 patients the supervising radiologist or urological input decided on a CT protocol, which included intravenous contrast. A further 21 patients had a previous history of calculi, and these were excluded, leaving 209 NECT KUB studies. In total, 269 CECT KUB scans were requested in 2020, and 55 scans were omitted either due to being a non-contrast enhanced study for reasons such as contraindication to iodinated contrast or following review and protocolling by senior radiologist. Therefore, 214 CECT studies were suitable for inclusion. No other exclusion criteria were applied. A total of 423 CT KUB studies were included (Figure 1).

### 2.3. Data Collection

The original radiological reports of the 423 studies were reviewed on the radiology information system (RIS). Collected data included gender, age and clinical details, which were provided on the imaging request form.

Referral symptoms were categorised into nephroureteric colic, abdominal pain or other. Those classified as abdominal pain did not have colic specified in their CT request or clinical features such as classical loin pain with radiation to the groin. Categorisation as other included those with lower urinary tract symptoms; haematuria; back pain; and symptoms suggestive of infection such as fever, rigors etc.

Other radiological findings identified (either possible alternative diagnoses or truly incidental) reported were recorded. The categorisation of these findings was carried out according to the Royal College of Radiologists (RCR) system for the reporting of incidental findings when conducting imaging research, and a study published by Lumbreras at al. (Supplementary S1) [16]. This system defines findings as either major, moderate or minor relative to their clinical significance and subsequent need for intervention or follow-up.

### 2.4. Imaging Acquisition

CT scans were all performed on 64-slice multidetector row scanners, either a GE Medical System Discovery CT750 HD or GE LightSpeed VCT (GE Medical System, Waukesha, Milwaukee, WI, USA). The scanning parameters were 120 kV, 40–120 mA, noise index of 58 for the NECT KUB and 120 kV, 130–280 mA, and noise index of 39 for CECT KUB. Contrast-enhanced studies received 50–100 mL of iodinated contrast intravenously.

### 2.5. Radiation Dose

In addition to the clinical demographic and imaging data, the radiation dose was recorded from each scan as per the original dose report. The dose was recorded as the dose length product (DLP) in mGy* cm. Standardised conversion factors were used to calculate the effective dose (EffD) in mSv of each case. A kappa-value of 0.015 (mSv mGy-1cm-1) as per the most updated ICRP was used [17].

### 2.6. Statistical Analysis

Data compilation and statistical analysis were performed using Microsoft Excel 2011 (Microsoft Corporation, Redmond, WA, USA) and Statistical Package for the Social Sciences version 28 (IBM, Chicago, IL, USA).

Continuous data were summarised as means and standard deviations. Baseline characteristics and outcomes were compared across both study groups with the use of chi-squared tests for presenting symptom and imaging diagnosis.

Key performance metrics including calculus detection rate (CDR), alternative CT findings, and negative studies were compared with the RCR standards for the whole group and for gender and age specified subgroups. Direct comparison was conducted between NECT and CECT for these subgroups and within these subgroups for gender using Chi-squared or Fishers Exact Test where applicable.

Differences in radiation dose exposure between techniques were compared using the *t*-test. Results with a *p* value < 0.05 were considered significant, and all testing was two-tailed.

## 3. Results

### 3.1. Baseline Characteristics

The two study groups were equally matched for both gender and mean age (Table 1). There was a significant difference in the age range between groups (*p* = 0.02). On comparing the presenting complaints between groups, a significant difference existed (*p* < 0.001) where a higher percentage of patients receiving CECT KUB demonstrated nephroureteric colic (77.6% vs. 55.5%).

### 3.2. Key Performance Indicators of Imaging

The calculus detection rate (CDR) for each whole group exceeded the RCR standard of >44%, with a CDRs of 56% and 54%, respectively, for NECT and CECT KUB (*p* = 0.643). Isolated alternative findings were present in 15% of NECT and 24% of CECT (*p* = 0.021). Additionally, there were dual findings in 7% of NECT and 14% of CECT (*p* = 0.045). A combination of dual and isolated alternative findings resulted in 23% of NECT and 40% of CECT having an abnormality identified. Imaging was negative in 28% of NECT and 22% of CECT (*p* = 0.168) (Table 2).

### 3.3. Presenting Complaints and Diagnosis

#### 3.3.1. Calculus Detection

In the NECT group, symptoms of colic were specified on the request form in 116 patients. Of these, 70 patients had calculi detected (60%). In total, 93 patients had no colicky symptoms described; however, 47 had a calculus detected (51%). Although the CDR appears higher in the cohort of patients with symptoms of colic, this was not statistically significant (*p* = 0.156).

In the CECT group, 166 patients had symptoms of colic specified on their request forms, with 111 of these having a calculus detected (66%). 48 patients in this group did not describe colicky symptoms; however, 3 had a calculus detected (8%). In contrast to the NECT cohort, patients undergoing a CECT had a significantly greater CDR when symptoms of renal colic were present (*p* < 0.001) (Supplementary S2.1).

#### 3.3.2. Alternative CT Findings

In the NECT group, 19% of those with symptoms other than renal colic and 26% of those with symptoms of renal colic were found to have alternative diagnosis. The higher rate of alternative diagnosis detection in those with renal colic symptoms was not statistically significant (*p* = 0.266).

In the CECT group, 48% of those without symptoms had alternative findings on imaging, while 37% who described renal colic had alternative findings. Similar to the NECT group, those with or without symptoms of renal colic did not have different rates of alternative diagnosis detection (*p* = 0.188) (Supplementary S2.2).

### 3.4. Subgroup Analysis of Key Performance Indicators: Gender and Age-Specific

#### 3.4.1. Alternative CT Findings

There were alternative findings (isolated or in combination with calculi) in 20% of NECT studies and 36% of CECT’s in the male cohort (*p* = 0.003). In females, NECT had a 29% alternative pick up, with CECT having a 44% rate (*p* = 0.04). When comparing imaging with alternative findings between males and females for NECT and CECT, no significant difference exists (*p* = 0.12 and 0.229, respectively).

#### 3.4.2. Calculus Detection

The gender-specific CDR over each age range between techniques can be seen in Supplementary S3. The RCR standard of >44% was achieved in both techniques in all age ranges for males except for those >70 years who underwent NECT imaging. There was no statistically significant difference in the CDR for males when comparing between techniques (*p* = 0.52). In females who had imaging (NECT or CECT), the RCR standard was not met; the CDR was 42% in NECT and 34% in CECT. A difference in the detection of calculi in females between techniques for the whole group was not statistically significant (*p* = 0.31). When comparing the CDR between males and females for NECT and CECT, a significant difference exists with *p*-values of 0.002 and <0.001, respectively.

#### 3.4.3. Negative Studies

In males 23% of NECT studies had no abnormal findings compared to 14% of CECT studies (*p* = 0.067). Females had a normal NECT in 37% of cases and normal CECT in 33% (*p* = 0.637). When comparing imaging with no abnormal findings between males and females for NECT and CECT, a significant difference exists with *p*-values 0.027 and <0.001, respectively (Supplementary S4.1–S4.3).

### 3.5. Intergroup Analysis: Categorisation of Alternative Findings

**Figure 2:**
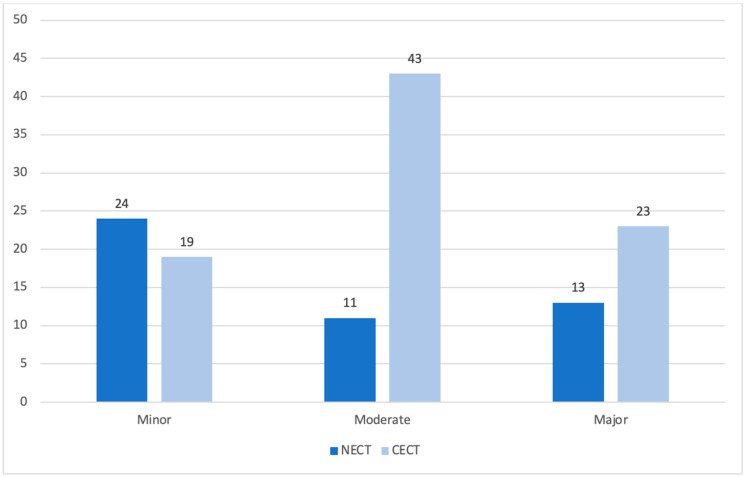
Bar chart categorising alternative findings as per RCR between NECT and CECT KUB. The bar chart highlights the number of minor, moderate and major alternative findings found in the NECT cohort (dark blue) and CECT cohort (light blue). The numbers above each bar represent the number of findings within that category.

There were 48 alternative findings in the entire NECT group (including both males and females). Of these, 13 were major, 11 were moderate and 24 were minor. The anatomical system breakdown was 19 reno-adrenal, 8 gynaecologic, 7 peritoneal, 5 musculoskeletal, 4 hepatobiliary and 5 reticuloendothelial findings. In comparison, there were 85 alternative findings in the entire CECT group (including both males and females), of which 23 were major, 43 were moderate and 19 were minor (Figure 2). The anatomical system breakdown of these findings was 34 reno-adrenal, 10 gynaecologic, 13 peritoneal, 6 musculoskeletal, 12 hepatobiliary, 7 reticuloendothelial and 1 vascular finding. On comparing the severity classifications, there was a statistically significant difference between the NECT and CECT cohorts (*p* < 0.001).

There was radiological evidence of pyelonephritis in 6 cases, simple renal cysts in 10, a ureteric and bladder mass (separate cases), and pelvicalyceal obstruction in the reno-adrenal system on NECT imaging. Outside of the reno-adrenal system there were three cases of appendicitis; a pre-sacral mass; an acute vertebral fracture; a suspicious pulmonary nodule; ovarian vein thrombosis; seven adnexal cysts (three complex, four simple); and two cases of splenomegaly, along with several other findings.

**Figure 3:**
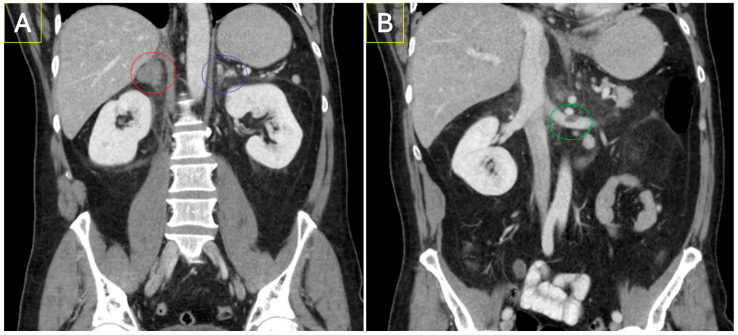
Two coronal slices of a CECT KUB of 58-year-old male who presented with symptoms of nephroureterolithiasis. NECT KUB imaging was normal, and patient was observed on ward and treated with analgesia; 48 h later, the patient developed adrenal crisis and subsequently had an IV enhanced CT. A right adrenal haematoma (red circle) and left adrenal haemorrhage (blue circle) are seen in (**A**), while (**B**) demonstrates a left renal vein thrombosis (green circle) that was unreported on index NECT KUB.

In CECT studies, ten cases had evidence of pyelonephritis, one had renal abscess, one case had pyelitis and three complex renal cysts were visualised. There were a few suspicious findings within the reno-adrenal system; an adrenal mass, a renal mass, a ureteric lesion, and prostatomegaly with localised lymphadenopathy. Other reno-adrenal findings included PUJ obstruction, adrenal nodules, adrenal haemorrhage (Figure 3), hydrocoele, hydronephrosis, adrenal myelolipomas, and evidence of cystitis. Major findings elsewhere included acute appendicitis, acute diverticulitis, acute cholecystitis, acute vertebral fracture, sclerotic bony lesions, oesophageal and bowel thickening with respective regional lymphadenopathy, splenic infarct, unexplained bulky lymphadenopathy, complex adnexal cysts, and hydrosalpinx. Acute colitis and pancreatitis, splenomegaly and indeterminate liver lesions were other common moderately classified findings. A complete list of described diagnoses for the NECT and CECT groups can be seen in Supplementary S5.1 and S5.2.

### 3.6. Radiation Dose Data

The mean DLP for the NECT group was 243.5 ± 109.2 mGy.cm, conferring an EffD of 3.645 ± 1.64 mSv. The CECT group had a significantly greater radiation exposure with an average and standard deviation (SD) DLP of 580.6 ± 172.3 mGy.cm and a corresponding EffD of 8.70 ± 2.58 mSv (*p* < 0.001). CECT was, on average, 2.4 times the radiation dose exposure of a non-enhanced study.

## 4. Discussion

This study observed the added utility of intravenous contrast administration in CT KUB investigations for suspected NU calculi from ED referrals. NECT KUB is the current gold standard technique in adequately resourced centres worldwide, with sensitivities and specificities for calculi reported in the range of 96–100% [1,2,12]. Institutionally, a 50% rise in demand for CT KUB imaging over 5-years led to concern over the limitations of the gold standard investigation in patients without calculi. The ethical and potential medico-legal consequences of missed findings heralded the change in protocol from NECT to CECT. The aim of the study was to observe the additional diagnostic output with CECT and accompanying radiation exposure and compare diagnostic equivalence regarding calculi detection.

Certainly, CECT resulted in an increased number of alternative findings than NECT. Major findings were almost doubled and moderate findings quadrupled, while minor findings were lower on CECT. Isolated alternative findings in the CECT group represented 25% of presentations and a further 15% for those with findings in addition to calculi. Therefore, cumulatively, 40% of those undergoing CECT had some form of alternative finding, with this figure being emphatically outside of the RCR 6–18% recommendation. Several acute emergencies including acute appendicitis, diverticulitis, pancreatitis and cholecystitis were identified. In addition, more indolent suspicious lesions in the adrenals, kidneys, ureters, liver and gastrointestinal tract were also identified. Whether truly incidental or possibly contributory to presentation, clearly a high proportion are clinically relevant, requiring further intervention. Of course, definite incidentals contributed to these proportions, the majority of which included simple adnexal cysts, hepatic steatosis and cholelithiasis. Sarofim et al. followed up 215 NECT KUBs and found that 33.5% of patients had other radiological findings, with 4% requiring surgery and 3% further imaging. Of the findings described as incidental by Sarofim et al., the majority are classified as minor in the current study [10]. Common overlapping incidentals reported included simple cystic lesions (adnexal, renal), cholelithiasis, fibroids and non-specific isolated lymphadenopathy. These appear to be ubiquitous after any form of CT KUB imaging, with a true incidental rate being reported as ~14% [18].

Although the CECT and NECT groups were unequally matched with differences in their age ranges and presenting symptoms, the CDR was maintained across both groups. Thus, we can infer retrospectively that CECT did not perform inferiorly to NECT when considering its primary objective of identifying stones. Other studies have also confirmed the non-inferiority of CECT at stone detection [19]. An assumption that those with non-colicky symptoms described would lead to alternative diagnosis rather than calculi was not found. Patients who had alternative findings on their CT presented with an arbitrary distribution of dichotomised symptoms, i.e., colic or non-colic.

Females had a CDR below the RCR recommendations regardless of technique (i.e., CECT or NECT). Males, however, had persistently high CDR’s > 60% across both techniques with neither being superior to the other. These findings indicate that, regardless of contrast administration, males and females tend to have high and low CDRs, respectively. This means that contrast neither impeded nor improved detection rates. This poor performance of CT KUB in the female population has been previously reported [12,13,14]. This phenomenon is also apparent in the negative studies in the female group, with numbers remaining stubbornly high across the NECT and CECT groups (37% and 33% respectively). Indeed, this cohort remains a diagnostic dilemma when presenting to the ED with symptoms suggestive of NU.

All CT imaging carries the risks associated with ionising radiation exposure. The UK National Dose Reference Level (DRL) for NECT KUB examinations addressing suspicion of NU is a DLP of 460 mGy.cm. This equates to an EffD of 6.44 mSv [15]. The DRL for CECT AP imaging (both oral and intravenous) is 745 mGy.cm (EffD-10.43 mSv). Thus, a significant increase in radiation exposure is associated with contrast-enhanced studies. The average EffD conferred by CECT at this institution was 8.70 ± 2.58 mSv. These patients have exceeded the DRL by ~25% because of protocol change. IV contrast enhancement has resulted in a significantly increased number of findings, and while many of these may be completely incidental, a significant proportion will require further medical intervention, either surveillance, diagnostic or therapeutic. The remaining question is what proportion of these findings on CECT would not have been identifiable on NECT, and this has yet to be answered.

A limitation of our study is that varied patients were included in the NECT and CECT cohorts, and there was a lack of ground truth. Although selection criteria remained the same (i.e., suspicion of NU), the variation in the cohorts made comparisons between the performance of the two modalities difficult. A prospective comparative study in patients with a known diagnosis (i.e., the availability of ground truth) would address this limitation and allow for the assessment of sensitivity and specificity of NECT versus CECT for stone detection and alternative finding detection.

It must be emphasised that current evidence does not advocate for CECT use in every case of suspected NU. Our study has shown that patients are exposed to significantly greater levels of ionising radiation with CECT compared to NECT. Therefore, despite the greater alternative diagnosis rate seen with CECT, its use needs to be limited to avoid unnecessary exposure of radiation to patients for whom NECT would have sufficed. In order to determine which patients should be selected for CECT over NECT, a direct comparative may be necessary.

This would clarify whether a hybrid approach of selectively using CECT, balancing the risk of increased radiation exposure with the added benefit of increased alternative findings, is possible. A simple approach would be to perform NECT on patients with a high pre-test probability of NU while reserving CECT for those with a low pre-test probability. One research group has suggested NECT for patients with a known history of NU and positive urinalysis and CECT for those with no prior NU history and a negative urinalysis [20].

Although a prospective direct comparative study in the same patients would be ideal, it would require a 2-phase scan and high levels of ionising radiation exposure. A more feasible option for future research may be a prospective randomised study using age-, gender- and symptom-matched patients. Once a definitive answer on CECT versus NECT utilisation in suspected NU is reached, a clinical decision support tool may be helpful in optimising modality use and workflow in the emergency department.

## 5. Conclusions

CECT results in a significantly increased number of both major and moderate findings other than calculi in ED referrals with suspected NU when compared retrospectively to NECT. Many but not all may be causative of presentation but regardless have some clinical relevance, i.e., a need for further medical intervention. Females presenting to the ED with symptoms suggestive of renal pathology are a group that remain a difficult to diagnose despite the use of IV CECT KUB. Exposure to ionising radiation is of significant concern, and CECT confers a 2.4-fold increase over NECT KUB.

## Figures and Tables

**Table 1:** Baseline characteristics of the study participants.

Characteristic	NECT KUB n = 209	CECT KUB n = 214	*p*-Value
**Age Mean (Std.Dev)**	45 (±16.8)	46.4 (±13.1%)	0.329
**Age Range**			
≤19	15 (7.2%)	3 (1.4%)	0.02
20–29 years	25 (12%)	15 (7%)	
30–39 years	40 (19.1%)	50 (23.4%)	
40–49 years	51 (24.4%)	55 (25.7%)	
50–59 years	43 (20.6%)	59 (27.6%)	
60–69 years	16 (7.7%)	20 (9.3%)	
70–79 years	14 (6.7%)	10 (4.7%)	
≥80	5 (2.4%)	2 (0.9%)	
**Presenting Symptom-no. (%)**			
Nephroureteric colic	116 (55.5%)	166 (77.6%)	<0.001
Abdominal pain	80 (38.3%)	45 (21%)	
Other	13 (6.2%)	3 (1.4%)	

**Table 2:** Key performance indicators between groups.

Key Performance Metrics	NECT KUB N = 209	CECT KUB N = 214	*p*-Value
Calculus Detection Rate	117 (56%)	115 (54%)	0.643
Calculus Only	101 (48%)	82 (38%)	0.038
Other Findings Only	32 (15%)	52 (25%)	0.021
Dual Findings	16 (7%)	33 (14%)	0.045
Negative	58 (28%)	47 (22%)	0.168

## Data Availability

Data is available via the corresponding author upon reasonable request. Although anonymised, data has been collected from a single institution from a specific cohort of patients (cystic fibrosis patients). Therefore, patients may be identifiable, and data cannot be made publicly available.

## Supplementary Materials

The following supporting information can be downloaded at: https://www.mdpi.com/article/10.3390/diagnostics15141731/s1, Supplementary S1: Classification of incidental findings on CT; Supplementary S2.1: Association with presenting symptom and calculus detection; Supplementary S2.2: Association with presenting symptom and alternative finding; Supplementary S3. Key performance indicators between NECT and CECT for gender and age-range; Supplementary S4.1 Alternative findings frequency and percentage table; Supplementary S4.2: Calculus detection frequency and percentage table; Supplementary S4.3: Negative studies frequency and percentage table; Supplementary S5.1. Alternative findings on CECT (n = 85); Supplementary S5.2 Alternative findings on NECT (n = 48).

## Abbreviations

The following abbreviations are used in this manuscript:

NU	Nephroureterolithiasis
ED	Emergency department
IVP	Intravenous pyelogram
NECT KUB	Non-enhanced computed tomography of the kidneys, ureters, and bladder
IV	Intravenous
RCC	Renal cell cancers
RCR	Royal College of Radiology
CDR	Calculus detection rate
CECT	Contrast enhanced computed tomography
EffD	Effective dose
DLP	Dose length product
