# Supplementary material for: Performance of Computed Tomography of the Kidneys, Ureter and Bladder in Non-Calculus Diagnoses: A Comparative Review of Non-Enhanced with Intravenous Contrast-Enhanced Imaging
Source: Diagnostics (Basel). 2025 Jul 8;15(14):1731. doi: 10.3390/diagnostics15141731 (PMC12293321; doi:10.3390/diagnostics15141731)
Supplement: Supplementary file 1 [file diagnostics-15-01731-s001.zip › Supplementary 1.pdf]

Supplementary 1.1 Classification of incidental findings on CT

Head-chest

- **Major:**
  - Parietal meningioma
  - Orbital mass
  - Parotid mass
  - Severe foraminal stenosis
- **Moderate:**
  - Chiari malformation
  - Circle of Willis calcifications
  - Mastoiditis
  - Thyroid incidentalomas
- **Minor:**
  - Arachnoid cyst
  - Large cisterna magna
  - Hürthle cell adenoma
  - Follicular adenoma
  - Parathyroid adenoma

Vascular

- **Major:**
  - Aortic aneurysm
  - Thoracic aneurysm
  - Iliac artery aneurysm
  - Thrombus
  - Common femoral artery pseudoaneurysm
  - Dissecting aorta
- **Moderate:**
  - Abdominal aortic ectasia
  - Pulmonary artery dilatation
  - Signs of portal venous hypertension
  - Atherosclerosis
  - Hepatic or vertebral haemangioma
  - Coronary artery calcification
  - Iliac artery ectasia
  - Rectus muscle haemangioma
- **Minor:**
  - Left-sided vena cava
  - Retroaortic left renal vein
  - Vascular graft

Reticuloendothelial

- **Major:**
  - Lymphadenopathy
  - Abdominal lymph node >1 cm
- **Moderate:**
  - Splenomegaly

- **Minor:**
  - Splenic cyst
  - Abdominal lymph node <1 cm

Hepatobiliary

- **Major:**
  - Solid hepatic mass
  - Solid pancreatic mass
  - Indeterminate liver lesion ≥1 cm
  - Indeterminate pancreatic lesion ≥1 cm
- **Moderate:**
  - Common bile duct dilatation
  - Gallstone
  - Hepatomegaly
  - Indeterminate hepatic lesion
  - Liver cirrhosis
  - Pancreatic calcifications
  - Pancreatic mass
  - Pancreatitis
  - Mild pancreatic duct dilatation
- **Minor:**
  - Calcified hepatic or splenic granulomas
  - Cholelithiasis
  - Hepatic cysts
  - Hepatic steatosis
  - Pancreatic head cyst
  - Small perihepatic fluid collection
  - Indeterminate liver lesion <1 cm
  - Hepatic haemangioma

Gynaecological

- **Major:**
  - Ovarian teratoma
  - Complex ovarian or adnexal cyst
  - Post-menopausal endometrial thickening
- **Moderate:**
  - Breast nodule
  - Uterine enlargement
- **Minor:**
  - Uterine fibroids
  - Simple ovarian cyst
  - Uterine calcifications
  - Bartholin's cysts

Musculoskeletal

- **Major:**
  - Vertebral body deformation suspected destruction
  - Lytic bone lesion

    - Indeterminate sclerotic bone lesion
- **Moderate:**
- **Minor:**
    - Degenerative spine changes
    - Pigmented villonodular synovitis
    - Spondylolisthesis
    - Diffuse osteopenia
    - Sclerotic bone lesion, likely bone island
    - Spina bifida occulta
    - Osteoarthritis

Peritoneal cavity

- **Major:**
    - Appendicitis
    - Indeterminate retroperitoneal masses
    - Pelvic mass
    - Ascites
    - Indeterminate soft-tissue mass in abdominal wall
    - Ileal wall thickening
- **Moderate:**
    - Abdominal wall hernia
    - Pelvic fluid collection
- **Minor:**
    - Appendiceal stone
    - Umbilical hernia
    - Hiatal, ventral, umbilical, or Bochdalek's hernia

Renoadrenal

- **Major:**
    - Adrenal mass with indeterminate appearance
    - Hydronephrosis with marked parenchymal reduction
    - Renal mass
    - Severe bilateral renal parenchymal reduction
    - Suspected undescended testis
    - Gallbladder wall thickening
    - Soft-tissue density within the gallbladder
- **Moderate:**
    - Adrenal adenoma
    - Adrenal mass with benign appearance
    - Hydronephrosis
    - Indeterminate adrenal nodule
    - Prostate enlargement
    - Renal angiomyolipoma
    - Renal parenchymal reduction
    - Solitary kidney
    - Pyelonephritis
    - Urethra-pelvic junction obstruction
    - Bladder outlet obstruction
    - Complex renal cyst

    - Scrotal hydrocoele
- **Minor:**
    - Adrenal myelolipoma
    - Bladder diverticulum
    - Bladder stone
    - Gallbladder absent or not seen
    - Mild renal parenchymal reduction
    - Renal atrophy
    - Renal calculi
    - Renal cyst
    - Renal malrotation
    - Small renal calcifications
    - Suspected renal stones
    - Suspected ureteric stone

Gastrointestinal tract

- **Major:**
    - Bowel obstruction
    - Gastric mass
    - Terminal ileum mass or thickening
    - Bowel wall thickening
- **Moderate:**
    - Hyperplastic colonic polyp
    - Bowel inflammation
    - Diverticulosis
    - Inguinal hernia or bowel-containing abdominal hernia
- **Minor:**
    - Hiatal hernia
    - Diaphragmatic hernia
    - Focal gastritis
    - Gastric fundus diverticulum
    - Rectal inflammation and/or haemorrhoids

Thoracic cavity

- **Major:**
    - Cardiomegaly
    - Idiopathic pulmonary fibrosis
    - Pneumothorax
    - Pulmonary embolism
- **Moderate:**
    - Bronchiectasis
    - Pericardial effusion
    - Pneumobilia
    - Pulmonary nodules
    - Pulmonary parenchymal opacity
    - Consolidation and infiltrates
    - Interstitial lung disease
    - Pleural fluid
    - Pulmonary emphysematous bullae

    - Mitral annulus calcifications
    - Tracheomalacia
- **Minor:**
    - Calcified pulmonary nodules
    - Pleural plaques
    - Subcutaneous emphysema
    - Lung base subsegmental atelectasis, scarring, and dependent changes
    - Diaphragmatic calcification
    - Cystic lung lesion
    - Pericardial granuloma

Others

- **Minor:**
    - Splenic, pulmonary, hepatic or adrenal granuloma
    - Lipoma
    - Findings in orthodontic panoramic radiographs: radio-opacities, thickening of mucosal lining in sinus, periapical inflammatory lesion, dentigerous cyst, cyst within alveolar bone, odontoma, altered tooth morphology, marginal bone loss
