# Supplementary material for: Performance of Computed Tomography of the Kidneys, Ureter and Bladder in Non-Calculus Diagnoses: A Comparative Review of Non-Enhanced with Intravenous Contrast-Enhanced Imaging
Source: Diagnostics (Basel). 2025 Jul 8;15(14):1731. doi: 10.3390/diagnostics15141731 (PMC12293321; doi:10.3390/diagnostics15141731)
Supplement: Supplementary file 1 [file diagnostics-15-01731-s001.zip › Supplementary 2.pdf]

Supplementary 2.1: Association with presenting symptom and calculus detection

			Calculus			
			Yes	***No***	***Total***	***P-value***
**NECT**	**Colic**	***Yes***	70	46	116	0.156
		No	47	46	93	
		Total	117	92	209	
**CECT**	**Colic**	***Yes***	111	55	166	<0.001
		No	4	44	48	
		Total	115	99	214	

Supplementary 2.2: Association with presenting symptom and alternative finding

			Alternative finding			
			Yes	***No***	***Total***	***P-value***
**NECT**	**Colic**	***Yes***	30	86	116	0.266
		No	18	75	93	
		Total	48	161	209	
**CECT**	**Colic**	***Yes***	62	104	166	0.188
		No	23	25	48	
		Total	85	129	214	
