# Supplementary material for: Performance of Computed Tomography of the Kidneys, Ureter and Bladder in Non-Calculus Diagnoses: A Comparative Review of Non-Enhanced with Intravenous Contrast-Enhanced Imaging
Source: Diagnostics (Basel). 2025 Jul 8;15(14):1731. doi: 10.3390/diagnostics15141731 (PMC12293321; doi:10.3390/diagnostics15141731)
Supplement: Supplementary file 1 [file diagnostics-15-01731-s001.zip › Supplementary 3.pdf]

Supplementary 3. Key performance indicators between NECT and CECT for gender and age-range

Age group	NECT	CECT	P-value	NECT	CECT	P-value	NECT *P-value	CECT *P-value
	Male alternative findings			*Female alternative findings*			*Between Male and Female*	
**≤19**	22%	100%	0.300	0%	50%	0.25	0.486	1
**20-29**	15%	40%	0.533	17%	50%	0.10	0.93	1
**30-39**	12%	26%	0.196	27%	42%	0.35	0.237	0.23
**40-49**	28%	31%	0.784	37%	48%	0.47	0.517	0.212
**50-59**	10%	39%	0.006	42%	43%	0.92	0.016	0.726
**60-69**	36%	33%	1.000	40%	45%	1.00	1	0.582
**70-79**	20%	25%	0.145	50%	0%	0.47	0.52	0.444
**≥80**	50%	100%	1.000	0%	N/A	N/A	0.4	N/A
**Total**	20%	36%	0.003	29%	44%	0.04	0.12	0.229
	Male calculus detection rate			*Female calculus detection rate*			*Between Male and Female*	
**≤19**	56%	100%	1.00	50%	0%	0.464	1	0.333
**20-29**	54%	60%	0.814	42%	30%	0.571	0.182	0.329
**30-39**	72%	71%	0.932	27%	42%	0.350	0.013	0.043
**40-49**	75%	66%	0.412	47%	39%	0.591	0.046	0.052
**50-59**	71%	61%	0.397	75%	30%	0.012	0.791	0.022
**60-69**	55%	78%	0.279	40%	36%	1.00	0.59	0.064
**70-79**	30%	88%	0.015	0%	0%	N/A	0.505	0.067
**≥80**	0%	50%	1.00	0%	N/A	N/A	N/A	N/A
**Total**	64%	68%	0.518	42%	34%	0.311	0.002	<0.001
	Male negative study			*Female negative study*			*Between Male and Female*	
**≤19**	22%	0%	1.00	50%	50%	1.00	0.329	1
**20-29**	38%	20%	0.615	42%	20%	0.277	0.87	1
**30-39**	16%	19%	0.745	47%	26%	0.218	0.035	0.564
**40-49**	16%	19%	0.740	21%	35%	0.327	0.623	0.178
**50-59**	19%	11%	0.345	8%	39%	0.056	0.38	0.011
**60-69**	18%	0%	0.479	60%	27%	0.299	0.245	0.218
**70-79**	50%	0%	0.019	50%	100%	0.467	1	0.022
**≥80**	50%	0%	1.00	100%	0%	N/A	0.4	N/A
**Total**	23%	14%	0.067	37%	33%	0.637	0.027	<0.001
