# Supplementary material for: Performance of Computed Tomography of the Kidneys, Ureter and Bladder in Non-Calculus Diagnoses: A Comparative Review of Non-Enhanced with Intravenous Contrast-Enhanced Imaging
Source: Diagnostics (Basel). 2025 Jul 8;15(14):1731. doi: 10.3390/diagnostics15141731 (PMC12293321; doi:10.3390/diagnostics15141731)
Supplement: Supplementary file 1 [file diagnostics-15-01731-s001.zip › Supplementary 4.pdf]

Supplementary 4.1 Alternative findings frequency and percentage table.

	NECT									CECT							
	Total	Male			Female			Within		Total	Male			Female			Within
**Age group**	**(n)**	**N**	**Alternative findings**	**%**	**n**	**Alternative findings**	**%**	**P-value**	**Age group**	**(n)**	**n**	**Alternative findings**	**%**	**n**	**Alternative findings**	**%**	**P-value**
**≤19**	15	9	2	22%	6	0	0%	0.486	≤19	3	1	1	100%	2	1	50%	1
**20-29**	25	13	2	15%	12	2	17%	0.93	20-29	15	5	2	40%	10	5	50%	1
**30-39**	40	25	3	12%	15	4	27%	0.237	30-39	50	31	8	26%	19	8	42%	0.23
**40-49**	51	32	9	28%	19	7	37%	0.517	40-49	55	32	10	31%	23	11	48%	0.212
**50-59**	43	31	3	10%	12	5	42%	0.016	50-59	59	36	14	39%	23	10	43%	0.726
**60-69**	16	11	4	36%	5	2	40%	1	60-69	20	9	3	33%	11	5	45%	0.582
**70-79**	14	10	2	20%	4	2	50%	0.52	70-79	10	8	2	25%	2	0	0%	0.444
**≥80**	5	2	1	50%	3	0	0%	0.4	≥80	2	2	2	100%	0	0	N/A	N/A
	209	133	26	20%	76	22	29%	0.12		214	124	45	36%	90	40	44%	0.229

Supplementary 4.2: Calculus detection frequency and percentage table.

	NECT									CECT									
	Total	Male			Female			Within		Total	Male			Female			Within	Between (Male)	Between (Female)
**Age group**	**(n)**	**n**	**Calculi**	**CDR**	**N**	**Calculi**	**CDR**	**P-value**	**Age group**	**(n)**	**n**	**Calculi**	**CDR**	**n**	**Calculi**	**CDR**	**P-value**	**P-value**	**P-value**
**≤19**	15	9	5	56%	6	3	50%	1.0	≤19	3	1	1	100%	2	0	0%	0.333	1.00	0.464
**20-29**	25	13	7	54%	12	5	42%	0.182	20-29	15	5	3	60%	10	3	30%	0.329	0.814	0.571
**30-39**	40	25	18	72%	15	4	27%	0.013	30-39	50	31	22	71%	19	8	42%	0.043	0.932	0.35
**40-49**	51	32	24	75%	19	9	47%	0.046	40-49	55	32	21	66%	23	9	39%	0.052	0.412	0.591
**50-59**	43	31	22	71%	12	9	75%	0.791	50-59	59	36	22	61%	23	7	30%	0.022	0.397	0.012
**60-69**	16	11	6	55%	5	2	40%	0.590	60-69	20	9	7	78%	11	4	36%	0.064	0.279	1.00
**70-79**	14	10	3	30%	4	0	0%	0.505	70-79	10	8	7	88%	2	0	0%	0.067	0.015	N/A
**≥80**	5	2	0	0%	3	0	0%	N/A	≥80	2	2	1	50%	0	0	N/A	N/A	1.00	N/A
	209	133	85	64%	76	32	42%	0.002		214	124	84	68%	90	31	34%	<0.001	0.518	0.311

Supplementary 4.3: Negative studies frequency and percentage table.

	NECT									CECT									
	Total	Male			Female			Within		Total	Male			Female			Within	Between (Male)	Between (Female)
**Age group**	**(n)**	**n**	**No findings**	**%**	**n**	**No findings**	**%**	**P-value**	**Age group**	**(n)**	**n**	**No findings**	**%**	**n**	**No findings**	**%**	**P-value**	**P-value**	**P-value**
**≤19**	15	9	2	22%	6	3	50%	0.329	≤19	3	1	0	0%	2	1	50%	1	1	1
**20-29**	25	13	5	38%	12	5	42%	0.87	20-29	15	5	1	20%	10	2	20%	1	0.615	0.277
**30-39**	40	25	4	16%	15	7	47%	0.035	30-39	50	31	6	19%	19	5	26%	0.564	0.745	0.218
**40-49**	51	32	5	16%	19	4	21%	0.623	40-49	55	32	6	19%	23	8	35%	0.178	0.74	0.327
**50-59**	43	31	6	19%	12	1	8%	0.38	50-59	59	36	4	11%	23	9	39%	0.011	0.345	0.056
**60-69**	16	11	2	18%	5	3	60%	0.245	60-69	20	9	0	0%	11	3	27%	0.218	0.479	0.299
**70-79**	14	10	5	50%	4	2	50%	1	70-79	10	8	0	0%	2	2	100%	0.022	0.019	0.467
**≥80**	5	2	1	50%	3	3	100%	0.4	≥80	2	2	0	0%	0	0	0%	N/A	1	N/A
	209	133	30	23%	76	28	37%	0.027		214	124	17	14%	90	30	33%	<0.001	0.067	0.637
