# Supplementary material for: Performance of Computed Tomography of the Kidneys, Ureter and Bladder in Non-Calculus Diagnoses: A Comparative Review of Non-Enhanced with Intravenous Contrast-Enhanced Imaging
Source: Diagnostics (Basel). 2025 Jul 8;15(14):1731. doi: 10.3390/diagnostics15141731 (PMC12293321; doi:10.3390/diagnostics15141731)
Supplement: Supplementary file 1 [file diagnostics-15-01731-s001.zip › Supplementary 5.pdf]

Supplementary 5.1. Alternative findings on CECT (n = 85)

System	RCR Classification	Radiological Finding	System	RCR Classification	Radiological Finding
**Reno-adrenal**	Major	Renal mass: Suspicious of RCC	**Peritoneal**	Major	Acute diverticulitis
	Major	Ureteric lesion		Major	Acute diverticulitis
	Major	Prostatomegaly with prominent iliac nodes and pulmonary nodule		Major	Acute appendicitis
	Major	Adrenal mass: Indeterminant		Major	Thickening of ascending colon and adenopahy
	Moderate	Prostatitis, cystitis, epididymitis		Major	Esophageal thickening with lymphadenopathy
	Moderate	Prostatitis, cystitis, epididymitis		Moderate	Prominent ileocolic nodes
	Moderate	Pyelonephritis		Moderate	Colitis
	Moderate	Pyelonephritis		Moderate	Colitis
	Moderate	Pyelonephritis		Moderate	Colitis
	Moderate	Pyelonephritis		Moderate	Colitis
	Moderate	Pyelonephritis		Moderate	Inguinal stranding with hernia
	Moderate	Pyelonephritis		Minor	Epiploic appendagitis
	Moderate	Pyelonephritis		Minor	Mesenteric panniculitis
	Moderate	Pyelonephritis	**Musculoskeletal**	Major	Acute vertebral fracture
	Moderate	Pyelonephritis		Major	Sclerotic lesion in vertebral body
	Moderate	Pyelonephritis		Major	Sclerotic lesion in femur
	Moderate	Renal abscess		Minor	Erosive sacroiliitis
	Moderate	Pyelitis		Minor	Fibrous dysplasia of ilium
	Moderate	PUJ obstruction		Minor	Old T11 compression fracture
	Moderate	PUJ obstruction	**Hepatobiliary**	Major	Acute cholecystitis
	Moderate	Bilateral adrenal nodules		Major	Acute cholecystitis
	Moderate	Adrenal nodule		Moderate	Acute pancreatitis
	Moderate	Adrenal nodule		Moderate	Acute pancreatitis
	Moderate	Complex renal cyst		Moderate	Pancreatic duct dilatation
	Moderate	Complex renal cyst		Moderate	Liver lesion: Indeterminant
	Moderate	Complex renal cyst		Moderate	Liver lesion: Indeterminant
	Moderate	Bilateral adrenal nodules		Moderate	Gallbladder polyp
	Moderate	Hydrocoele		Minor	Hepatic steatosis
	Moderate	Adrenal haemorrhage		Minor	Cholelithiasis
	Moderate	Ureteritis, cystitis		Minor	Cholelithiasis
	Moderate	Hydronephrosis		Minor	Pancreatic cyst
	Minor	Bilateral myelolipomas	**Reticuloendothelial**	Major	Splenic infarcts

	Minor	Cystitis		Major	Para-aortic lymphadenopathy
	Minor	Cystitis		Major	Mesenteric adenopathy
**Gynaecologic**	Major	Bilateral hydrosalpinx-> Later endometriosis confirmed		Major	Iliac fossa adenopathy
	Major	Adnexal cyst: Complex		Major	Splenomegaly + upper abdominal lymphadenopathy
	Major	Adnexal cyst: Large		Moderate	Splenic lesion: Indeterminant
	Major	Adnexal cyst: Complex + free fluid		Moderate	Splenomegaly
	Minor	Corpus luteal cyst		Moderate	Splenomegaly
	Minor	Corpus luteal cyst	**Other**	Minor	Calcified SMA aneurysm
	Minor	Uterine fibroid			
	Minor	Adnexal cyst: Simple			
	Minor	Adnexal cyst: Unspecified			
	Minor	Bartholin’s cyst			

Supplementary 5.2 Alternative findings on NECT (n = 48)

System	RCR Classification	Radiological Finding	System	RCR Classification	Radiological Finding
**Reno-adrenal**	Major	Pelvicalyceal obstruction	**Peritoneal**	Major	Pre-sacral mass
	Major	Bladder mass		Major	Appendicitis
	Major	Ureteric mass		Major	Appendicitis
	Moderate	Pyelonephritis		Major	Appendicitis
	Moderate	Pyelonephritis		Minor	Faecal loading
	Moderate	Pyelonephritis		Minor	Diverticula
	Moderate	Pyelonephritis		Minor	Diverticula
	Moderate	Pyelonephritis	**Musculoskeletal**	Major	Acute T12 fracture
	Moderate	Pyelonephritis		Moderate	Erosive sacroilitis
	Moderate	PUJ obstruction		Minor	Degenerative spine
	Minor	Renal cyst: Simple		Minor	Spondylolisthesis
	Minor	Renal cyst: Simple		Minor	Degenerative spine
	Minor	Renal cyst: Simple	**Hepatobiliary**	Moderate	Hepatic nodularity
	Minor	Renal cyst: Simple		Minor	Hepatic steatosis
	Minor	Renal cyst: Simple		Minor	Hepatic steatosis
	Minor	Renal cyst: Simple		Minor	Cholelithiasis
	Minor	Renal cyst: Simple	**Reticuloendothelial**	Major	Spiculated pulmonary nodule 7mm
	Minor	Renal cyst: Simple		Major	Ovarian vein thrombosis
	Minor	Renal cyst: Simple		Moderate	Splenomegaly
	Minor	Renal cyst: Simple		Moderate	Splenomegaly
**Gynaecologic**	Major	Haemorrhagic ovarian cyst		Minor	Localised lymphadenopathy
	Major	Adnexal cyst (complex)			
	Major	Adnexal cyst 6.7 cm			
	Minor	Adnexal cyst (simple)			

	Minor	Adnexal cyst 2.5 cm
	Minor	Adnexal cyst 4 cm
	Minor	Adnexal cyst 3.7cm
	Minor	Pelvic calcification
